# Association Between Changes in Oral Health-Related Quality of Life and Depressive Symptoms in the Korean Elderly Population

**DOI:** 10.3389/ijph.2023.1605403

**Published:** 2023-03-28

**Authors:** Kyoung Eun Park, Hooyeon Lee, Young Dae Kwon, Sukil Kim

**Affiliations:** ^1^ Department of Preventive Medicine, College of Medicine, The Catholic University of Korea, Seoul, Republic of Korea; ^2^ Department of Humanities and Social Medicine, College of Medicine and Catholic Institute for Healthcare Management, The Catholic University of Korea, Seoul, Republic of Korea

**Keywords:** depressive symptoms, aged, oral health related quality of life, geriatric oral health assessment index, Korean longitudinal study of ageing

## Abstract

**Objectives:** The aim of this study was to examine the association between changes in oral health related quality of life (OHRQoL) and depressive symptoms in the elderly South Koreans.

**Methods:** We used the 2018 and 2020 Korean Longitudinal Study of Ageing data. Our study population was a total of 3,604 participants aged over 65 in 2018. The independent variable of interest was the changes in the Geriatric Oral Health Assessment Index as OHRQoL between 2018 and 2020. The dependent variable was depressive symptoms in 2020. Multivariable logistic regression analyzed the associations between changes in OHRQoL and depressive symptoms.

**Results:** Participants with improvement in OHRQoL over 2-year period were likely to have fewer depressive symptoms in 2020. Especially, changes in the oral pain and discomfort dimension score was associated with depressive symptoms. A decline in oral physical function, such as difficulty in chewing and speaking, was also associated with depressive symptoms.

**Conclusion:** Negative change in OHRQoL is a risk factor for depression in elderly. This results suggest the importance of maintaining good oral health in later life, as a protective factor against depression.

## Introduction

According to the Organization for Economic Co-operation and Development (OECD) demography statistics, the average annual increase of South Korea’s elderly population was 3.3% from 1970 to 2018, which is the fastest among all OECD countries (1). In 2020, 15.7% of the Korean population was aged ≥65 years; this is expected to increase to 20.3% in 2025 and 43.9% in 2060 (2). As the aging population increases rapidly, the importance of oral health of the elderly is increasing. Oral health is an essential factor for maintaining health, wellbeing, and quality of life (3). The World Health Organization defines oral health as “a state of being free from mouth and facial pain, oral and throat cancer, oral infection and sores, periodontal (gum) disease, tooth decay, tooth loss, and other diseases and disorders that limit an individual’s capacity in biting, chewing, smiling, speaking, and psychosocial wellbeing” (4).

Previous studies have suggested an association between tooth loss and the incidence of depressive symptoms among older adults (5, 6). Depression is associated with untreated tooth decay, loss of natural teeth, and frequency of dental visits (7, 8). Some studies reported that reductions in subjective mastication and pronunciation ability, and oral dryness are risk factors for depression (9, 10). The deterioration of oral function and orofacial appearance related to social activities; speaking, smiling, and eating, significantly mediated the relationship between tooth loss and the incidence of depressive symptoms (6).

Oral health can be defined and measured both objectively and subjectively. Along with the number of remaining teeth and chewing ability, oral health-related quality of life (OHRQoL) has received considerable attention as a subjective marker of oral health. OHRQoL considers not only chewing ability, but also more information about several other important oral health parameters. Patient-centered care is becoming increasingly important. OHRQoL is a representative measure of subjective oral health that was developed to analyze the effects of self-perceived oral health on daily activities and wellbeing (11, 12).

Many studies have demonstrated that a lower OHRQoL is associated with a higher likelihood of depressive symptoms (5, 13–16). Longitudinal studies reported that worsening showed worsening in OHRQoL is associated with depressive symptoms at follow-up (5, 14). Similarly, cross-sectional studies conducted in Korea showed association between lower OHRQoL and higher depression in elderly populations (17). However, those studies included small study population or used cross-sectional study design. Therefore, this study aims to clarify the association between changes in OHRQoL and depressive symptoms in an elderly South Korean population using representative data and longitudinal analysis.

## Methods

### Data

We used 2018 and 2020 data from the Korean Longitudinal Study of Ageing (KLoSA), which provides in-depth interview data from Koreans aged ≥45 years living in households selected by multistage stratified probability sampling. The respondents were considered representative of the Korean adult population. Trained interviewers collected the data using a computer-aided interview system (18).

**FIGURE 1:**
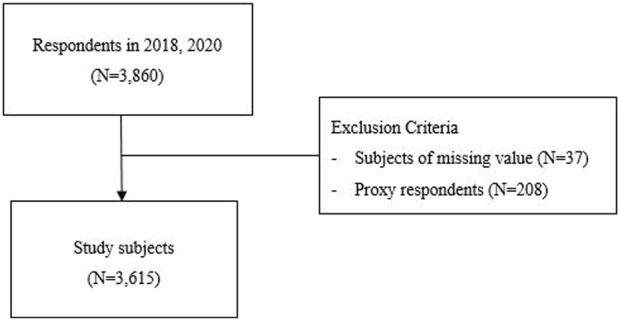
Selection of study subjects (Korean longitudinal study of ageing, South Korea, 2018–2020).

In total, 3,860 patients aged ≥65 years in 2018 participated in both the 2018 and 2020 surveys. Participants with missing data (*n* = 36) were excluded from the analysis. The questionnaires from proxy respondents (*n* = 208) were also excluded because their data on depressive symptoms and other indicators of psychological wellbeing were considered potentially unreliable (19). Ultimately, 3,615 elderly Koreans were included in our study. (Figure 1). This study was approved by the Institutional Review Board of The Catholic University of Korea (MC22ZISI0042).

### Variables

The independent variable was the changes in OHRQoL assessed using the Geriatric Oral Health Assessment Index (GOHAI) between 2018 and 2020. GOHAI is self-reported questionnaire designed to assess oral health problems affecting quality of life, especially for use in the elderly population (20). The GOHAI includes 12 questions related to oral health and the frequency of oral health problems. The problems are rated using a 6-point Likert scale (0, always; 1, very often; 2, often; 3, sometimes; 4, seldom; 5, never). The GOHAI is calculated by summing the scores of the 12 items; total scores thus range from 0 to 60. Higher scores indicate higher OHRQoL.

The GOHAI encompasses three dimensions: physical function dimension (limitation with certain food types, difficulty with biting/chewing, uncomfortable to swallow, prevented from speaking), psychosocial dimension (limited contact with other people, unhappy with appearance of gums and teeth, worried about teeth or gums, nervous/self-conscious about teeth or gums, uncomfortable eating in front of others), and pain and discomfort dimension (discomfort when eating, use of medications to relieve gum pain, teeth sensitive to hot/cold). We used a validated Korean translation of the GOHAI (21). The Cronbach’s alpha of the GOHAI was 0.830 in this study.

Dependent variable was the presence of depressive symptoms in 2020, as assessed by the Center of Epidemiologic Studies Depression Scale, 10-item version (CES-D10) (22). Total scores range from 0 to 10, and the cutoff CES-D10 score for the depressive symptoms is 4 (23). The Cronbach’s alpha for the CES-D10 was 0.853 in this study.

Covariates were included based on the results of previous population-based studies of depressive symptoms, including age (65–69, 70–74, 75–79, or ≥80 years), sex (male or female), educational level (elementary school or less, middle school, high school, or college or over), spouse (yes or no), number of family members (1, 2, or ≥3), health insurance type (National Health Insurance or medical aid), household income quartile (Q1–4), self-rated health (SRH) (good, fair, or poor), chronic diseases (0, 1–2, or ≥3), and activity of daily living (ADL) (0, 1 or over). Oral health was assessed according to changes in the numbers of dental implants and remaining teeth.

### Statistical Analysis

Sampling weights were applied to estimate the representative population. Categorical and continuous data are expressed as weighted percentages and means, respectively. The chi-square test and t-test were conducted to examine the distribution and differences in sociodemographic characteristics, health-related characteristics according to depressive status, and changes in GOHAI total, dimension, and item scores. Multivariable logistic regression analysis was performed to analyze the associations between changes in OHRQoL and depressive symptoms, adjusted by 2018 baseline characteristics (GOHAI, socioeconomic characteristics, and health-related characteristics) and changes in oral health (numbers of dental implants and using denture).

To prevent multicollinearity, separate multivariate logistic regression models were constructed to evaluate the total GOHAI scores and GOHAI subdimension scores. Statistical significance was set at α = 0.05. Data were analyzed using SAS ver. 9.4 (SAS Institute Inc., Cary, NC, USA).

## Results

### General Characteristics

Table 1 shows the distribution of the 2018 baseline general characteristics of the study subjects. Female was 57.1%, and the greatest proportion in educational level was elementary school or less 47.4%. Regarding living arrangements, 69.3% of the participants lived with their spouse and 56.2% lived with at least two family members. With respect to SRH, only 22.0% of the subjects self-rated their health status as “Good”. 21.2% did not have a chronic disease and 2.2% had ADL limitations.

**TABLE 1:** Baseline general characteristics of the study subjects in 2018 (Korean longitudinal study of ageing, South Korea, 2018–2020).

Variable	Category	Weighted (%)
Socioeconomic characteristics		
Sex	Male	(42.9)
	Female	(57.1)
Age, y	65–69	(35.3)
	70–74	(25.3)
	75–79	(20.7)
	≥80	(18.6)
Education level	Elementary school or less	(47.4)
	Middle school	(18.2)
	High school	(24.7)
	College or over	(9.6)
Spouse	Yes	(69.3)
	No	(30.7)
Living family members, n	1	(21.6)
	2	(56.2)
	≥3	(22.3)
Health insurance type	National health insurance	(94.4)
	Medical aid	(5.7)
Household income quartile (Q)	1Q	(20.7)
	2Q	(22.4)
	3Q	(26.6)
	4Q	(30.3)
Health-related characteristics		
Self-rated health	Good	(22.0)
	Fair	(47.8)
	Bad	(30.3)
Chronic diseases, n	0	(21.2)
	≤2	(56.3)
	≥3	(22.5)
ADL, n	0	(97.8)
	≥1	(2.2)

ADL, activities of daily living.

### Changes in OHRQoL and Oral Health

The GOHAI score was 37.5 ± 0.15 in 2018 and 38.3 ± 0.14 in 2020. OHRQoL was slightly higher in 2020 than 2018. Regarding the dimensions of the GOHAI, the physical function dimension and oral pain and discomfort dimension scores increased more than the psychosocial dimension score between 2018 and 2020. Issues related to chewing, speech, and oral pain and discomfort improved positively. The GOHAI items showing the greatest score increases were “Able to swallow comfortably” (physical function dimension), and “Able to eat without discomfort” and “Used medication to relieve pain” (pain and discomfort dimension).

We also analyzed oral health according to the number of remaining teeth and dental implants. The number of remaining teeth was 19.6 ± 0.17 in 2018 and 17.3 ± 0.19 in 2020, and the number of implants was 1.3 ± 0.06 in 2018 and 3.8 ± 0.13 in 2020. The number of remaining teeth decreased approximately by 2.3, while the number of dental implants increased approximately by 2.5. Using denture was 30.4% in 2018 and it decreased to 28.8% in 2020 (Table 2).

**TABLE 2:** Changes in oral health-related quality of life and oral health (Korean longitudinal study of ageing, South Korea, 2018–2020).

Variable	Weighted (%), weighted mean ± SE		p–value
	2018	2020	
GOHAI score (0–60) *	37.5 ± 0.15	38.3 ± 0.14	<.001
GOHAI dimension scores			
Physical function (0–20) *	12.8 ± 0.06	13 ± 0.06	0.002
Psychosocial function (0–25) *	15.4 ± 0.06	15.6 ± 0.06	0.018
Pain or discomfort (0–15) *	9.3 ± 0.04	9.7 ± 0.04	<.001
GOHAI item scores (0–5)			
Physical function			
Limitations in kinds of food	3.1 ± 0.02	3.1 ± 0.02	0.793
Trouble biting or chewing	3.1 ± 0.02	3.2 ± 0.02	0.512
Able to swallow comfortably*	3 ± 0.02	3.2 ± 0.02	<.001
Unable to speak clearly	3.5 ± 0.02	3.5 ± 0.02	0.353
Psychosocial function			
Limited contact with people	3.6 ± 0.02	3.6 ± 0.02	0.165
Pleased with appearance of teeth	1.9 ± 0.02	1.8 ± 0.02	0.804
Worried about gums or dentures*	3.3 ± 0.02	3.4 ± 0.02	0.003
Self-conscious about gums or dentures*	3.3 ± 0.02	3.4 ± 0.02	0.008
Uncomfortable eating in front of others	3.4 ± 0.02	3.4 ± 0.02	0.173
Pain or discomfort			
Able to eat without discomfort*	2.6 ± 0.02	2.8 ± 0.02	<.001
Used medication to relieve pain*	3.5 ± 0.02	3.6 ± 0.02	<.001
Sensitive to hot, cold, or sweet foods	3.2 ± 0.02	3.3 ± 0.02	0.057
Number of remaining teeth	19.6 ± 0.17	17.3 ± 0.19	<.001
Number of implants*	1.2 ± 3.3	3.7 ± 7.9	<.001
Use Denture			
Yes	(30.4)	(28.8)	0.187
No	(69.6)	(71.2)	

* *p*-value <0.05.

SE, standard error.

### Baseline Characteristics by 2020 Depressive Symptoms

Table 3 shows 2018 baseline characteristics by 2020 depressive symptoms. In total, 11.8% of the subjects had depressive symptoms. Female, aged ≥80 years, a lower educational level (elementary or less), a lower income level (Q1), and poor health status (poor SRH or chronic diseases) showed more proportion in depressive symptoms group. Especially the subjects with depression had poor oral health compared with those without depressive symptoms. The mean GOHAI score was 5.5 points lower in the depression group than in the group without depressive symptoms (32.6 ± 0.39 vs. 38.1 ± 0.15), indicating that the depressed patients had a lower OHRQoL. Furthermore, the mean number of teeth was 2.7 lower in the depression group than in the group without depressive symptoms (17.3 ± 0.53 vs. 20 ± 0.18), as was the mean number of implants (1.1 ± 0.14 vs. 1.4 ± 0.06). Finally, the proportion of patients with dentures was 7.1% higher in the depressive symptoms group than in the group without depressive symptoms (36.7% vs. 29.6%).

**TABLE 3:** Baseline characteristics according to depressive symptoms in 2020 (Korean longitudinal study of ageing, South Korea, 2018–2020).

Variable	Weighted (%) or weighted mean ± SE			*p*–value
	Depressive symptoms		No depressive symptoms	
Number of participants	(11.8)		(88.2)	
Oral health-related characteristics^a^				
GOHAI score (0–60)	32.6 ± 0.39		38.1 ± 0.15	<0.001
Physical function score (0–20)	10.7 ± 0.15		13 ± 0.06	<0.001
Psychosocial function score (0–25)	13.7 ± 0.19		15.6 ± 0.07	<0.001
Pain or discomfort score (0–15)	8.2 ± 0.1		9.5 ± 0.04	<0.001
Number of remaining teeth	17.3 ± 0.53		20 ± 0.18	<0.001
Number of implants	1.1 ± 0.14		1.4 ± 0.06	0.105
Use denture^b^				
Yes	(36.7)		(29.6)	<.0001
No	(63.3)		(70.4)	
Socioeconomic characteristics^b^				
Sex				
Male	(36.2)		(43.8)	<0.001
Female	(63.8)		(56.2)	
Age, y				
65–69	(25.6)		(36.6)	<0.001
70–74	(22.2)		(25.7)	
75–79	(21.8)		(20.6)	
≥80	(30.5)		(17.1)	
Education level				
Elementary school or less	(57.2)		(46.1)	<0.001
Middle school	(15.0)		(18.6)	
High school	(20.7)		(25.3)	
College or over	(7.2)		(10.0)	
Spouse				
Yes	(59.9)		(70.5)	<0.001
No	(40.1)		(29.5)	
Living family members, n				
1	(28.6)		(20.6)	<0.001
2	(47.9)		(57.3)	
≥3	(23.5)		(22.1)	
Health insurance type				
National health insurance	(89.2)		(95.0)	<0.001
Medical aid	(10.8)		(5.0)	
Household income quartile (Q)				
1Q	(31.4)		(19.3)	<0.001
2Q	(21.1)		(22.6)	
3Q	(23.4)		(27.0)	
4Q	(24.2)		(31.2)	
General health-related characteristics^†^				
Self-rated health				
Good	(10.6)		(23.5)	<0.001
Fair	(37.6)		(49.1)	
Bad	(51.8)		(27.4)	
Chronic diseases, n				
0	(12.7)		(22.3)	<0.001
≤2	(54.5)		(56.6)	
≥3	(32.8)		(21.1)	
ADL				
0	(93.2)		(98.4)	<0.001
≥1	(6.8)		(1.6)	

^a^ Analyzed by t-test.

^b^ Analyzed by chi-square test.

SE, standard error; ADL, activities of daily living.

### Association Between Changes in OHRQoL and Depressive Symptoms

Compared with unadjusted odds ratio in model 1, the likelihood of depressive symptoms was significantly associated with changes in OHRQoL (OR: 0.92, 95% CI: 0.92–0.92) after adjustment for 2018 baseline characteristics and changes in oral health. It indicates increase in GOHAI score was associated with lower risk of depressive symptoms at follow-up.

In model 2, changes in the oral pain and discomfort dimension score (OR: 0.86, 95% CI: 0.79–0.93) had the strongest association with depressive symptoms; changes in physical function score (OR: 0.90, 95% CI: 0.85–0.95) was also significantly associated with depressive symptoms. It indicates increase on the oral pain and discomfort and physical function GOHAI subdimensions were inversely associated with the likelihood of depressive symptoms at follow-up. However, there were no associations between the changes in psychosocial subdimension scores and depressive symptoms at follow-up. Thus, improved oral pain and discomfort and physical function were associated with fewer depressive symptoms in our elderly Korean population (Table 4).

**TABLE 4:** Association between changes in oral health-related quality of life and depressive symptoms (Korean longitudinal study of ageing, South Korea, 2018–2020).

Variable	Unadjusted OR (95% CI)	*p*–value	Adjusted OR (95% CI)	*p*–value
Model 1^a^				
Change in GOHAI score	0.98 (0.98–0.98)	<.001	0.92 (0.92–0.92)	<.001
Model 2^b^				
Change in physical function score	0.97 (0.97–0.97)	<.001	0.90 (0.85–0.95)	<.001
Change in psychosocial function score	0.96 (0.96–0.96)	<.001	0.99 (0.94–1.04)	0.680
Change in pain or discomfort score	0.92 (0.92–0.93)	<.001	0.86 (0.79–0.93)	<.001

^a^ OR adjusted for 2018 characteristics (GOHAI, age, sex, education level, spouse, living family members, health insurance type, household income, self-rated health, chronic disease, ADL) and changes in oral health (number of dental implants, using denture or not).

^b^ OR adjusted for 2018 characteristics (GOHAI physical function score, psychosocial function score, pain or discomfort score, age, sex, education level, spouse, living family members, health insurance type, house hold income, self-rated health, chronic disease, ADL) and changes in oral health (number of dental implants, using denture or not).

GOHAI, Geriatric Oral Health Assessment Index; ADL, activities of daily living; OR, odds ratio; CI, confidence interval.

## Discussion

This study analyzed the association between changes in OHRQoL, as assessed by the GOHAI, and depressive symptoms in elderly (aged ≥65 years) South Koreans. Participants with improved OHRQoL over 2-year period were likely to have fewer depressive symptoms in 2020, especially when oral pain and discomfort dimension changed positively, the likelihood of depressive symptoms decreased the most. Our results are similar to those of previous studies. A longitudinal study conducted in the UK reported that deterioration in oral health and OHRQoL in an elderly cohort had a negative effect on depressive symptoms over a 4-year period (5). A Japanese study showed a low baseline OHRQoL increased the probability of depressive symptoms after 4 years (14). Also, a cross-sectional study reported that lower OHRQoL was associated with more severe depression in the elderly (15, 16). Caution is required when making comparisons with cross-sectional studies.

In this study, the prevalence of depressive symptoms was 11.8% in 2020, similar to the rates in other studies of the elderly (i.e., 9.1–9.3% (24) and 11.0% (14)). The GOHAI was 37.0 and 37.8 in 2018 and 2020 in our study, compared with 45.8 in a Mexican study, and 53.6 (15) and 50.8 (25) in Japanese studies. Differences in GOHAI among countries can be explained by differences in self-rated oral health status. In one study, South Koreans had poorer self-rated oral health compared with a US population, even though the oral health (i.e., the number of teeth; decayed, missing, filled teeth index; decayed teeth index; filled teeth index; and missing teeth index) considered better (26).

Differences between countries are more pronounced for the dimension of the GOHAI. A study in Japan, oral dryness and pain were associated with depression (13). A previous study in South Korea reported similar results; specifically, there was association between oral pain and physical dimension of the OHRQoL and depression (27). According to an Asian study of the elderly, oral pain and discomfort appears to be the most relevant dimension with respect to depression in elderly Asian populations (28).

A decline in oral physical function, such as difficulty in chewing and speaking, is also associated with depression (9). Difficulty chewing tough foods may have an impact on daily enjoyment and life in older adults. Eating with relatives and friends also involves communication. Decreased oral function can impair social contact in older adults (29).

Common causes of oral pain and discomfort in the elderly include dental caries (30), periodontal disease (31), and oral dryness (32), while problems with oral physical function arise due to unsuitable dentures, lost teeth, and oral dryness (32–34). To solve those problems, the Korean government has implemented insurance policies covering two implants, one partial or full denture treatment, and the costs of maintaining dentures (through the National Health Insurance plan for the elderly, i.e., those aged ≥65 years) (35). These healthcare policies have improved dental care for the Korean elderly (36) and could help reduce oral health-related depression in this age group. Therefore, clinicians managing elderly patients with depression should focus on oral health. The government should also consider oral health and depression together when developing mental health-promoting strategies for the elderly. This study provides basic data that could inform policies aimed at the prevention and management of depression in the elderly.

### Strengths and Limitations

This study had several limitations. First, we only analyzed changes in OHRQoL over a 2-year period; research considering longer periods is needed to confirm a causal relationship between oral health and depressive symptoms. Nevertheless, this study provides useful information for future long-term longitudinal studies. Second, the KLoSA data is secondary, and various oral health parameters potentially important to depression, such as gingival bleeding, oral dryness, and dissatisfaction with dentures, were not considered. Despite its limitations, this study showed an association between changes in OHRQoL and depressive symptoms based on an analysis of KLoSA data representative of the Korean elderly population.

### Conclusion

This study showed that decrease in Oral health-related quality of life was a risk factor for depression in elderly. Oral health is a dynamic phenomenon influenced by many factors that change over time, and can generate positive, as well as negative emotions. This results have important implications for gerontologists and practitioners. The importance of maintaining good oral health in later life, as a protective factor against depression.

## Abbreviations

CES-D10, Center of Epidemiologic Studies Depression Scale, 10-item version; CI, Confidence Interval; GOHAI, Geriatric Oral Health Assessment Index; KLoSA, Korean Longitudinal Study of Ageing; OECD, Organization for Economic Co-operation and Development; OHRQoL, Oral Health Related Quality of Life; OR, Odds Ratio; SRH, Self Rated Health; VIF, Variance Inflation Factor.
